# Comparative mitochondrial proteomic, physiological, biochemical and ultrastructural profiling reveal factors underpinning salt tolerance in tetraploid black locust (*Robinia pseudoacacia L*.)

**DOI:** 10.1186/s12864-017-4038-2

**Published:** 2017-08-22

**Authors:** Qiuxiang Luo, Mu Peng, Xiuli Zhang, Pei Lei, Ximei Ji, Wahsoon Chow, Fanjuan Meng, Guanyu Sun

**Affiliations:** 1 College of Life Science, Northeast Forestry University, Harbin, 150040 China; 2 Key Laboratory of Saline-alkali Vegetation Ecology Restoration in Oil Field (SAVER), Ministry of Education, Alkali Soil Natural Environmental Science Center (ASNESC), Northeast Forestry University, Harbin, China; 3 Division of Plant Science, Research School of Biology, The Australian National University, ACT, 2601 Australia

**Keywords:** Diploid, Mitochondria, Physiological characters proteomics, Salinity stress, Tetraploid black locust (*Robinia pseudoacacia L.*), Ultrastructure

## Abstract

**Background:**

Polyploidy is an important phenomenon in plants because of its roles in agricultural and forestry production as well as in plant tolerance to environmental stresses. Tetraploid black locust (*Robinia pseudoacacia L.*) is a polyploid plant and a pioneer tree species due to its wide ranging adaptability to adverse environments. To evaluate the ploidy-dependent differences in leaf mitochondria between diploid and tetraploid black locust under salinity stress, we conducted comparative proteomic, physiological, biochemical and ultrastructural profiling of mitochondria from leaves.

**Results:**

Mitochondrial proteomic analysis was performed with 2-DE and MALDI-TOF-MS, and the ultrastructure of leaf mitochondria was observed by transmission electron microscopy. According to 2-DE analysis, 66 proteins that responded to salinity stress significantly were identified from diploid and/or tetraploid plants and classified into 9 functional categories. Assays of physiological characters indicated that tetraploids were more tolerant to salinity stress than diploids. The mitochondrial ultrastructure of diploids was damaged more severely under salinity stress than that of tetraploids.

**Conclusions:**

Tetraploid black locust possessed more tolerance of, and ability to acclimate to, salinity stress than diploids, which may be attributable to the ability to maintain mitochondrial structure and to trigger different expression patterns of mitochondrial proteins during salinity stress.

**Electronic supplementary material:**

The online version of this article (doi:10.1186/s12864-017-4038-2) contains supplementary material, which is available to authorized users.

## Background

Salinity is a major limiting factor that adversely affects plant growth and crop productivity and quality worldwide [1]. Increased salinization may lead to a loss of 10 million ha of farming land each year [2]. In general, salinity stress may lead to water deficits, ion toxicity, osmotic stress, membrane alterations, ionic toxicity, and free radical production of plants [3]. Many plants exhibit slow growth or death under salinity stress. To survive this salinity stress, plants have evolved complex mechanisms based on modifications in metabolites, gene expression, and proteins [4]. To date, many genes responding to salinity stress in plants have been identified [5]. However, relatively little is known about the detailed physiological and molecular mechanisms underlying polyploid plants to salinity stress.

Polyploidization occurs in 75% angiosperms and 95% ferns in plants [6]. Therefore, polyploidy is a common phenomenon in the evolution of angiosperms [7, 8], which can facilitates adaptive evolution [9]. Polyploid plants play important roles in agricultural production due to their specific traits such as longer leaves [10], morphological enlargements in seeds and corolla limbs [11] and delayed flowering [12]. Additionally, some polyploidy forest species also showed a lot of advantages over their diploid counterparts, including larger leaves, greater growth vigour, better timber quality and higher stress resistance [13, 14]. However, empirical data supporting this hypothesis are scarce, especially for forest species [15].

In addition, many polyploids are superior to diploids with respect to tolerance to environmental stresses. In recent years, many studies have been conducted on polyploid plants. However, most of these studies are focused on various crops species, such as potato (*Solanum tuberosum*) [16], maize (*Zea mays* L.) [17], cotton (*Gossypium hirsutum*) [18] and cabbage (*Brassica oleracea* L) [19] or the model plant *Arabidopsis thaliana* [6, 20]. However, very little is known about polyploid forest species.

Generally, polyploidy can be classified as autopolyploidy from the doubling of chromosomes of a single species and allopolyploidy from the hybrids between two species. Tetraploids are a common type among polyploids [21]. As a polyploid plant, tetraploid black locust (*Robinia pseudoacacia* L.), which is native to Korea, is a preferred tree species in the timber forest due to its rapid growth and good wood texture. Generally, leaf size is double in 4× plants than 2× plant leaves. Moreover, it can be used as fine feed for domestic fowl and livestock because its fleshy leaves are rich in vitamins and minerals. Importantly, tetraploid black locust is a pioneer tree species due to its wide ranging adaptability to adverse environments such as salt, drought, cold, and infestation. Our earlier studies have shown that the mitochondria of tetraploid black locust can maintain a relatively intact ultrastructure compared with diploids under salinity stress [22, 23], which could be attributed to the nature of divergent genomes.

Proteomics is an effective method to assess the complete proteome at the tissue or organelle level to compare the effects of salinity stress is by salinity stress [24–27]. Two-dimensional polyacrylamide gel electrophoresis (2-DE) is one of the most powerful and sensitive techniques, and it has been applied to salt treatments for different plant species [28]. Based on the 2-DE method, it has been found that salt-tolerant genotypes are associated with a more abundant energy supply, higher reactive oxygen species (ROS) scavenging and ethylene production, and stronger photosynthetic capacity than salt-sensitive genotypes under salinity stress [25]. Nevertheless, the precise molecular mechanism underlying the role of plant mitochondria in resisting salinity stress is still unknown.

Plant mitochondria are involved in multiple metabolic and biosynthetic processes, and are especially important in the generation of adenosine triphosphate (ATP). In addition, mitochondria export organic acid intermediates for wider cellular biosynthesis and the photorespiratory pathway [29]. Particular, mitochondria play an important role in plant survival during stress [30]. To date, a number of studies have investigated the effects of abiotic stresses on plant mitochondria, which include salinity [31, 32], heat [33], drought and cold [34]. Among these stresses, salinity is a serious threat to plant growth that significantly alters plant metabolism, development and even leads to plant death. Some reports have also shown that salinity stress has a severe negative influence on mitochondrial respiration [35]. In fact, respiratory metabolism plays a key role in mediating plant tolerance to salinity [36]. The relationship between mitochondrial function and salinity tolerance has been investigated in some plant species, such as wheat [37], barley [36] and *Arabidopsis* [33, 38]. These studies have provided a good overview of the mitochondrial response to salinity stress. However, there is relatively little information available on polyploid plants. It is necessary to gain a better knowledge of the underlying mechanisms to understand how polyploid plants perform under salinity stress.

Here, to evaluate ploidy-dependent differences in leaf mitochondria between diploid (abbreviated as 2×) and tetraploid (abbreviated as 4×) black locust under salinity stress, we isolated and purified mitochondria from leaves, and compared the effects of salinity stress on mitochondria. Finally, the physiological, ultrastructural and proteomic traits of mitochondria in leaves of 2× and 4× black locust under salinity stress were investigated, and functional implications are discussed.

## Methods

### Plant materials and salt treatment

All materials were introduced directly from South Korea to China by Beijing Forestry University. The 2× and 4× black locust (*Robinia pseudoacacia* L.) were from one germplasm. Thus, they possess the same genetic origin. Thirty uniform plants (2 years old) of 2× and 4× were planted in plastic pots (21 cm in diameter and 21 cm in depth) filled with a 2:1 (*v*/v) mixture of soil and sand. The experiments were carried out at Harbin Experimental Forest Farm Greenhouse of Northeast Forestry University in June 2013. Potted plants were grown in the greenhouse (day/night air temperature, 28/22 °C; photoperiod, 12 h; relative humidity, 65–85%). Plants were treated with 250 (moderate salinity stress) or 500 mM NaCl (high salinity stress) for 7 days. After 7 days of treatment, the fully expanded fresh leaf tissues (the third to fifth leaves from the shoot apex) were collected for physiological measurements and transmission electron microscopy analysis. Other additional leaves were used for mitochondria extraction. At least three independent biological experiments for each treatment were replicated.

### Transmission electron microscopy

Fresh leaves about 1.5 cm^2^ in size were sampled, fixed immediately with 2.5% (*v*/v) glutaral pentanedial at 4 °C for 2 h, and washed twice in 0.1 M PBS (sodium phosphate buffer, pH 6.8) at 4 °C. Then they were post fixed in 2% osmium tetraoxide (O_s_O_4_) for 2 h, sequentially dehydrated by 50%, 70%, 90%, and 100% acetone, and embedded in Epon 812 for 2 h. Ultrathin sections (70 nm) were sliced, stained with uranyl acetate and lead citrate, and then mounted on copper grids for viewing on the H-600 IV TEM (Hitachi, Tokyo, Japan) at an accelerating voltage of 60 kV.

### Respiratory measurements

Leaf respiration rate was determined by a Clark-type oxygen electrode (Hansatech Instruments, Britain) [39]. 1 cm^2^ leaf segments were incubated in the dark for 30 min to minimize wounding effects and light-enhanced dark respiration. They were kept in a 2 ml reaction medium containing 10 mM HEPES, 10 mM MES (pH 7.2) and 2 mM CaCl_2_ and then the total respiration rate was determined. The rates of SHAM-resistant respiration (Cyt pathway) and KCN-resistant respiration (alternative pathway) were measured in the presence of SHAM (200 mM) and KCN (200 mM), respectively.

### Physiological and biochemical investigations

#### Measurements of Hydrogen peroxide (H_2_O_2_) and Malondialdehyde (MDA) contents

H_2_O_2_ was detected spectrophotometrically according to I Sergiev, V Alexieva and E Karanov [40]. A 0.5 ml mitochondrial extraction was homogenized in 5 ml 0.1% TCA on an ice bath. After centrifuged at 12,000×*g* for 10 min, 1 ml supernatant was mixed with 0.5 ml potassium phosphate buffer (pH 7.5) and 1 ml 1 M potassium iodide. The absorbance of supernatant was measured at 390 nm and the concentration of H_2_O_2_ was obtained using a standard curve.

MDA content was estimated by the method of Wang et al. [23] with some modifications. The extract was dissolved in 5 ml 10% TCA, centrifuged at 12,000×*g* for 10 min and the supernatant was transferred to a 5 ml centrifuge tube, diluted with 10% TCA to 4 ml. The supernatant (1 ml) was mixed with 4 ml 20% TCA containing 0.5% (*w*/*v*) thiobarbituric acid (TBA). The mixture was heated in boiling water for 15 min and immediately cooled on ice to stop the reaction; then the mixture was centrifuged at 12,000×g for 10 min. The absorbance of the final supernatant was measured at 532 nm, 600 nm and 450 nm. The MDA concentration was calculated by means of an extinction coefficient (155 mM^−1^ cm^−1^).

#### Measurements of enzyme activities

Superoxide dismutase (SOD, EC 1.15.1.1) activity was measured following the method of C Beauchamp and I Fridovich [41]. The reaction mixture contained 20 μL enzyme extract, 50 mM sodium phosphate buffer (pH 7.8), 100 μM EDTA, and 10 mM pyrogallol. Enzyme activity was detected at 420 nm by a spectrophotometer. Glutathione reductase (GR, EC 1.6.4.2) activity was determined by NADPH oxidation at 340 nm. The reaction mixture contained 10 μL enzyme extract, 100 mM potassium phosphate buffer (pH 7.8), 0.2 mM NADPH, 2 mM EDTA, and 0.5 mM glutathione. The reaction was initiated by adding NADPH at 25 °C [42]. Ascorbate peroxidase (APX, EC 1.11.1.11) activity assay was carried out by the method of Y Nakano and K Asada [43]. The reaction mixture contained 50 mM sodium phosphate buffer (pH 7) including 0.2 mM EDTA, 0.5 mM ascorbic acid, 50 mg BSA, and crude enzyme extract. The reaction was started by adding H_2_O_2_ at a final concentration of 0.1 mM. Cytochrome c oxidase (COX, EC 1.9.3.1) was determined according to the method of M Neuburger, EP Journet, R Bligny, JP Carde and R Douce [44]. Dehydroascorbate reductase (DHAR, EC 1.8.5.1) was measured following AsC formation at 265 nm in the reaction solution containing 0.5 mM DHA, 5 mM reduced glutathione (GSH) [45]. Monodehydroascorbate reductase (MDHAR, EC 1.6.5.4) activity was determined as NADH oxidation at 340 nm. The reaction mixture contained 0.2 mM NADH, 1 mM AsC, and 1 unit of AsC oxidase [46].

#### Measurements of ascorbate (AsA) and GSH contents

AsA content was determined following MY Law, SA Charles and B Halliwell [47] with some modifications. The reaction mixture included 0.2 ml protein extract, 0.5 ml phosphate buffer (150 mM, pH 7.4), and 0.2 ml double distilled water. Then 0.4 ml α′-dipyridyl in 70% ethyl alcohol and 0.2 ml FeCl_3_ (3%) were added to the reaction solution. The mixtures were incubated at 40 °C for about 40 min. After centrifugation at 12,000×*g* for 10 min, the clear supernatant was collected and the change in absorbance at 525 nm was monitored.

The determination of GSH was carried out following the method of G Ellman [48]. The absorbance of reduced chromogen and 5,5′-Dithiobis (2-nitrobenzoic acid) (DTNB) was measured at 412 nm and GSH concentration was determined.

### Two-dimensional gel electrophoresis

#### Isolation of mitochondria and estimation of mitochondrial protein

For isolation and purification of mitochondria from leaves, we used a method of [49] with slight modifications. All extraction procedures were carried out between 0 and 4 °C. Leaf tissue (30 g) was ground with pre-refrigerated pestle and mortar in 200 ml grinding media containing 50 mM HEPES (pH 7.5), 5 mM hexanoic acid, 0.3% BSA (*w*/*v*), 0.3 M sucrose, 10 mM β-mercaptoethanol, 20 mM EDTA, 30 mM Na-ascorbate and 1% (*w*/*v*) PVP. After that the homogenates were squeezed through a 40 × 40 μm mesh nylon cloth and centrifuged at 4000×*g* for 10 min. The supernatant was centrifuged at 20,000×*g* for 10 min. The precipitate was suspended and washed twice in wash medium buffer A (20 mM HEPES, 330 mM sorbic alcohol, 10 mM NaCl,2 mM EDTA, 5 mM Na-ascorbate) and centrifuged at 10,000×*g* for 15 min. After centrifugation the precipitate was suspended in washing medium. The suspension was then loaded onto a sucrose gradient consisting of 3 ml: 8 ml: 6 ml: 6 ml, bottom to top, of 57%, 45%, 37% and 25% sucrose. The mixture was centrifuged for 1 h at 40,000×*g* and mitochondria were present as an opaque band at the 37%/45% interface. Then the mitochondrial band was collected, washed and centrifuged at 20,000×*g* for 15 min in medium buffer A.

Mitochondrial proteins were extracted by adding 0.7 ml of 10% acetone to a tube and then stored at 20 °C for 12 h. Then samples were centrifuged at 25,000×g for 15 min. The precipitate was washed with 80% and 100% cold acetone, respectively and centrifuged for 30 min. After centrifugation, the precipitate was vacuum dried. The dried powder was dissolved in an IEF buffer containing 7 mM urea, 2 mM urea, 40 μM dithiothreitol (DTT), 0.2% pharmalytes (pH 4–7) and 4% 3-[(3-cholamidopropyl)-dimethylammonio]-1-propane sulfonate (CHAPS). The protein solution was stored at −80 °C until use. Mitochondrial protein concentration was determined by the method of MM Bradford [50].

To estimate protein contaminant in our mitochondrial isolation, a specific antibody against chloroplast RbcL was used to assess mitochondria contamination because this organelle is the common contaminant of chloroplast protein preparations. Additionally, the signals from antibodies against AOX recognized proteins from the both mitochondrial protein fractions and the total protein extracts, were used to estimate the purity of the mitochondria fractions.

#### Gel electrophoresis and gel staining

400 μg protein samples were rehydrated in 250 μl protein rehydration solution and used for isoelectric focusing (IEF). Subsequently, the IPG strips (13 cm, pH 4–7) were incubated for 12 h at room temperature. The operation was followed a procedure consisting of 30 V for 14 h, 100 V for 1 h, 500 V for 1 h, 1000 V for 1 h, 8000 V for 1 h and 8000 V for 5 h. After IEF, gels were equilibrated in 10 ml equilibration buffer containing 6 M urea, 50 μM Tris-HCl (pH 8.8), 2% (*w*/*v*) SDS, 30% glycerol and 1% (*w*/*v*) dithiothreitol (DTT) for 15 min with 2.5% iodoacetamide instead of DTT for 15 min. The SDS-PAGE in the second dimension electrophoresis was conducted using 12.5% (*w*/*v*) polyacrylamide gel. After electrophoresis, the gels were stained with a coomassie brilliant blue R-250 solution containing 25% methanol, 8% acetic acid and 0.1% (*w*/*v*) CBB until protein spots were clearly visible.

#### Gel image analysis and Matrix-Assisted Time of Flight Mass Spectroscopy (MALDI-TOF-MS) analysis

Gel images were scanned using an ImageScanner III (GE Healthcare, Bio-Sciences, Uppsala, Sweden). Images were analyzed with ImageMaster 2D Platinum 7.0 software (Amersham Biosciences, Piscataway, NJ, USA, 2011). The average volume percent values were calculated from three technical replicates to represent the final volume percent values of each biological replicate. The experimental molecular weight (*Mw*) and isoelectric point (*pI*) of the protein spots were determined by 2-DE standards and interpolation of missing values on the IPG strips. Spots were quantified based on total density of the gels by the percentage volume. Significantly different spots, which were determined as *p* < 0.05 and a change of more than 2.5-fold in abundance, were considered to be differentially accumulated proteins, and they had to be consistently present in three replications.

Selected protein spots were excised, washed with 50% (*v*/v) acetonitrile in 0.1 M NH_4_HCO_3_, and dried at room temperature. Proteins were reduced with 1 mM DTT and 2 mM NH_4_HCO_3_ at 55 °C for 1 h and alkylated with 55 mM iodoacetamide in 25 mM NH_4_HCO_3_ in the dark at room temperature for 45 min. The gel pieces were thoroughly washed with 25 mM NH_4_HCO_3_, 50% ACN, 100% ACN, and dried. The proteins were digested in 10 ml modified trypsin (Promega, Madison, WI, USA) solution (1 ng/ml in 25 mM ammonium bicarbonate) during an overnight incubation at 37 °C. Digests were immediately spotted onto 600 mm anchorchips (Bruker Daltonics, Bremen, Germany). Spotting was achieved by pipetting 1 ml analyte onto the MALDI target plate in duplicate and then adding 0.05 ml of 20 mg/ml α-CHCA in 0.1% TFA/33% (*v*/v) ACN, which contained 2 mM ammonium phosphate. All samples were analyzed in the positive-ion reflection mode on a TOF Ultraflex II mass spectrometer (Bruker Daltonics, Billerica, United states). Each acquired mass spectra (m/z range 700–4000, resolution 15,000–20,000) was processed using FlexAnalysis v2.4 software (Bruker Daltonics, Bremen, Germeny, 2004). Proteins were identified with Mascot software (http://www.matrixscience.com) based on the mass signals to search for proteins in the SwissProt, NCBInr, and MSDB databases. In addition, we also combined with knowledge from literature, 9 functional categories were obtained by blasting against the NCBInr, Swissprot and UniProt database (http://www.ebi.uniprot.org). And the gene-ontology based on the functional annotation using DAVID web-server and KEGG pathway was carried out.

#### Statistical analyses

Statistical analyses were performed with SPSS 17.0 software (SPSS Inc., Chicago, IL, USA, 2009). All parameters are presented as mean ± standard error (SD) and were obtained from at least three replicates and analyzed using Duncan’s multiple range test or Student’s *t*-test. A *p*-value <0.05 was considered significant.

### Western blot analysis

To confirm the proteomic data, western blot analysis was carried out with equal amount of mitochondria protein including three specific antibodies (ATP synthase β subnunit (spot 220, ASB), Heat Shock Protein 60 (spot 149, HSP) and β-actin (Agrisera, Sweden)). Western blot analysis was carried out by the method of [51] with minor modification. In brief, equal amount of mitochondria protein of black locust leaves (2× and 4×) were resolved on 10% SDS-PAGE and transferred onto nitrocellulose membrane (GE Healthcare, UK). The membranes were blocked with TBST buffer (10 mM Tris-HCl, pH 7.5, 150 mM NaCl, 0.05% Tween 20) containing 5% milk overnight and then incubated with specific antibodies (Agrisera, Sweden) in TBST over night, including ATP synthase β subnunit (ASB), Heat Shock Protein 60 (spot 149, HSP) and β-actin. After washing 3 times, the membrane was incubated with goat-anti-rabbit IgG secondary antibody conjugated to HRP (KPL, USA) diluted 1:10,000 in TBST for 1 h. Proteins were detected with enhanced chemiluminescence (ECL) reagents (Agrisera, Sweden). Western blot analysis experiments were repeated at least three times, and the representative data are shown.

### Quantitative real time PCR

To investigate the relationship between the transcriptional and translational levels of salinity stress related genes, we employed qRT-PCR for 12 genes selected based on the proteomics results (Additional file 1: Table S1). Total RNA was isolated using a plant RNA extraction kit (Bioteke, China) and cDNA was synthesized from 1 μg of the total RNA with PrimeScript Reverse Transcriptase (Takara, Japan) according to the manufacturer’s instructions. Specific primer pairs for the selected genes were designed by comparing the nucleotide sequence of conserved region using BioEdit, Premier 5.0 and Oligo 6.0 (Additional file 2: Table S2). The qRT-PCR was performed using the SYBR Green Realtime PCR Master Mix (Toyobo, Japan) with Lightlycler480 (Roche, USA), based on semi-quantitative reverse transcription PCR (RT-PCR) results (Additional file 2: Table S2). The expression levels of the *β-actin* were used as an internal control (reference gene). Relative expression of the target genes was calculated using the comparative *C*t method.

### Statistical analyses

Statistical analyses were performed with SPSS 17.0 software (SPSS Inc. Chicago, IL, USA, 2009). All parameters are presented as mean ± standard error and were obtained from at least three replicates. Parameters were analyzed using Duncan’s multiple range test or Student’s t-test. A *p*-value <0.05 was considered significant.

## Results

### Physicochemical characteristics

#### Effect of salt treatment on leaf growth and ultrastructure

**Fig. 1:**
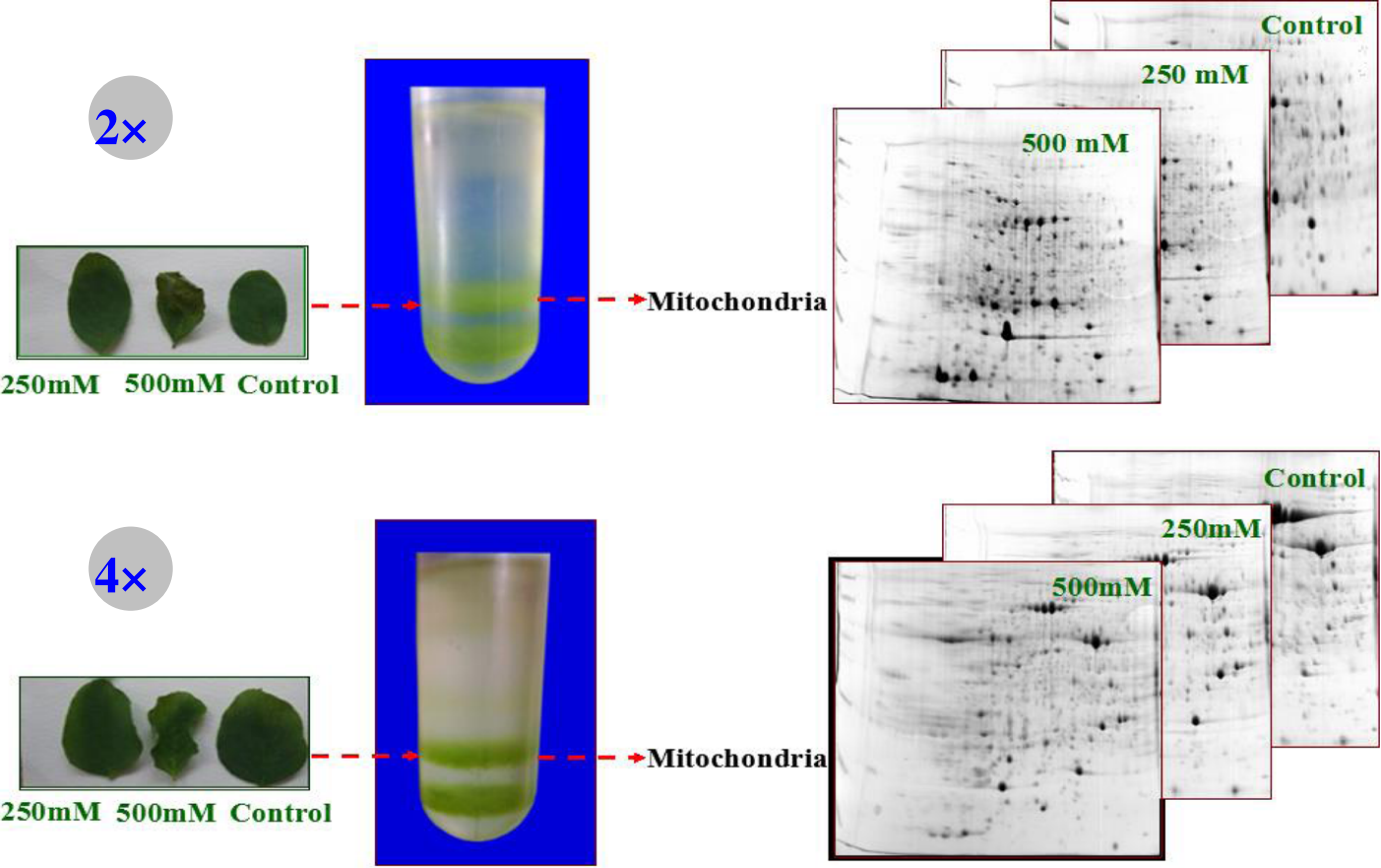
Images of leaves, extracted mitochondria and 2D gel under different NaCl concentrations (0, 250 and 500 mM)

The 2× leaves exhibited wilting from the leaf apex under 250 mM NaCl, as shown in Fig. 1. However, 4× leaves did not show obvious changes under the same conditions (Fig. 1). In addition, 500 mM NaCl inhibited 2× leaf growth (Fig. 1). In contrast, 4× leaves showed no serious damage under the same conditions (Fig. 1).

**Fig. 2:**
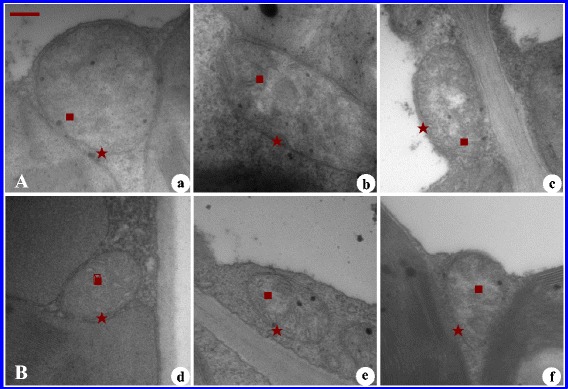
Ultrastructure of mitochondria of mesophyll cells of diploid (**a**, 2×) and tetraploid (**b**, 4×) *R. pseudoacacia* by transmission electron microscope (TEM) under 0, 250 mM and 500 mM NaCl treatment. Bar = 0.25 μm. **a** and **d**, Mitochondria of 2× and 4× under 0 mM NaCl, respectively; **b** and **e**, mitochondria of 2× and 4× under 250 mM NaCl; **c** and **f**, mitochondria of 2× and 4× under 500 mM NaCl. Five-pointed star (★), mitochondrial membrance; square (■), mitochondria cristae

Using leaf ultrastructure analysis, in our study we observed that salinity stress resulted in mitochondrial damage in leaves from 2× plants (Fig. 2). In control conditions, clear and intact membranes and cristae were observed in mitochondria of 2× and 4× leaves (Fig. 2a and d). In contrast, the changes in mitochondria from 4× leaves were relatively small under NaCl stress (250 mM NaCl and 500 mM NaCl) compared to 2× leaves (Fig. 2e and f). Some mitochondria in 2× leaves were almost devoid of cristae, and some mitochondrial membranes became invisible after NaCl treatment (Fig. 2b and c). In contrast, the relatively slight changes in mitochondria from 4× occurred under NaCl stress (Fig. 2e and f).

#### Effect of salt treatment on respiration rate

The rates of total respiration (*V*t), Cyt pathway respiration (*V*_Cyt_) and alternative pathway respiration (*V*_Alt_) in both 2× and 4× leaves increased after NaCl treatment (Table 1). In 2× leaves under 500 mM NaCl, the rates of *V*t, *V*_Cyt_ and *V*_Alt_ increased sharply by 3.80, 3.19 and 8.62 times, respectively, when compared with the control. Stressed 4× leaves showed similar responses to 2× leaves under NaCl stress. Interestingly, 4× leaves exhibited higher respiration rates (*V*t, *V*_Cyt_ and *V*_Alt_) compared to 2× leaves, under both 250 and 500 mM NaCl treatments.

**Table 1:** Effect of NaCl treatment on the respiration rates in leaves of diploid (2×) and tetraploid (2×) *R. pseudoacacia*

Treatment	Total respiration *V*t (nmol O_2_ g^−1^ FW s^−1^)	Cytochrome pathway respiration *V*_Cyt_ (nmol O_2_ g^−1^ FW s^−1^)	Alternative pathway respiration *V*_Alt_ (nmol O_2_ g^−1^ FW s^−1^)
2×			
0 mM	2.62 ± 0.025a	0.94 ± 0.035a	0.45 ± 0.050a
250 mM	5.98 ± 0.200b	2.33 ± 0.162b	2.24 ± 0.146b
500 mM	9.97 ± 0.510c	3.00 ± 0.265b	3.88 ± 0.278c
4×			
0 mM	3.49 ± 0.125a	0.88 ± 0.036a	0.77 ± 0.074a
250 mM	9.67 ± 0.495b	2.80 ± 0.127b	4.23 ± 0.104b
500 mM	13.59 ± 0.121c	4.80 ± 0.210c	5.34 ± 0.056b

Salt treatments were performed for 7 days. Values represent the means of three separate experiments ± standard deviations (SD)

#### Effects of salt treatment on H_2_O_2_ and MDA levels

**Fig. 3:**
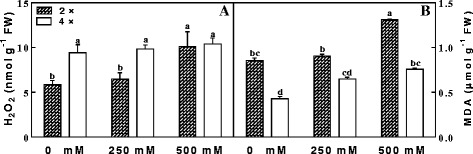
The contents of H_2_O_2_ (**a**) and MDA (**b**) in mitochondria of mesophyll cells of diploid (2×) and tetraploid (4×) *R. pseudoacacia* under 0, 250 mM and 500 mM NaCl treatment. Values represent the means of three separate experiments ± standard deviations (SD). Capital letters stand for significant difference at 5% level

To evaluate the adverse effects of NaCl stress on cell membranes, we measured the contents of H_2_O_2_ and MDA in mitochondria (Fig. 3). Compared with controls, no obvious changes were observed at high H_2_O_2_ levels of 4× samples after NaCl treatment. However, salinity induced an increases in H_2_O_2_ levels in 2× samples, compared to a relatively low value in controls. Meanwhile, MDA content increased in both 2× and 4× samples during the 7 days of stress, but MDA levels were higher in 2× samples than in 4× samples for each treatment, especially 500 mM NaCl treatment (Fig. 3). This result shows that mitochondria in 4× leaves could cope with salinity stress more effectively than in 2× leaves.

#### Effects of salt treatment on anti-oxidative enzyme activity and the AsA and GSH levels

**Fig. 4:**
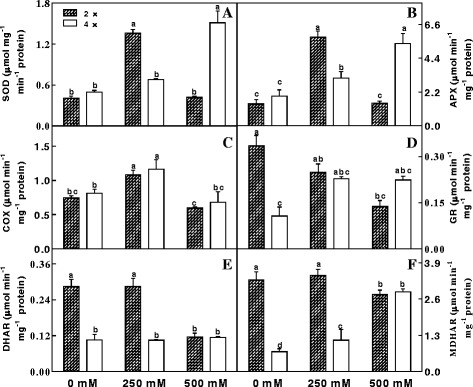
The activities of SOD (A), APX (B), COX (C), GR (D), DHAR (E), MDHAR (F) in mitochondria of mesophyll cells of diploid (2×) and tetraploid (4×) *R. pseudoacacia* under 0, 250 mM and 500 mM NaCl treatment. Values represent the means of three separate experiments ± standard deviations (SD). Capital letters stand for significant difference at 5% level

NaCl treatment (250 mM and 500 mM) induced significant increases in SOD and APX activities in 4× mitochondria. In contrast, SOD and APX activities in 2× mitochondria initially increased under 250 mM NaCl but then showed a marked drop under 500 mM NaCl (Fig. 4a and b). COX activity in 4× was slightly higher than in 2× after NaCl treatment (Fig. 4c). For GR activity, NaCl stress resulted in a significant decrease in 2×, but a pronounced increase in 4× (Fig. 4d). Meanwhile, DHAR activity in 4× showed little change after NaCl treatment. However, DHAR activity in 2× leaves initially changed little at 250 mM NaCl but then showed a marked drop at 500 mM NaCl (Fig. 4e). A similar trend was also observed in MDHAR activity in 2× leaves after NaCl treatment. In comparison with control leaves, 4 × leaves showed increased MDHAR activity after NaCl treatment (Fig. 4f).

**Fig. 5:**
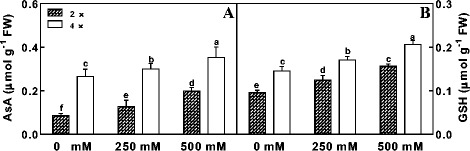
The contents of ASA (A) and GSH (B) in mitochondria of mesophyll cells of diploid (2×) and tetraploid (4×) *R. pseudoacacia* under 0, 250 mM and 500 mM NaCl treatment. Values represent the means of three separate experiments ± standard deviations (SD). Capital letters stand for significant difference at 5% level

NaCl treatment caused a significant increase in the levels of both ASA and GSH in 2× and 4× leaves (Fig. 5). Furthermore, the levels in 4× leaves were much higher than that in 2× leaves in each treatment (Fig. 5).

### Assessment of leaf mitochondria purity

**Fig. 6:**
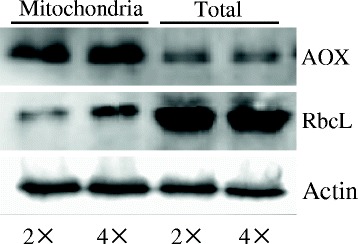
Western blot of alternative oxidase (AOX), ribulose-1,5-bisphosphate carboxylase/oxygenase (RbcL) and *β*-actin (Actin) in mitochondrial protein fraction (Mitochondria) and total protein (Total) extracts from controls of 2× and 4× leaves

As shown in Fig. 6, there was a weak band detected in the mitochondrial protein fractions, compared with the signals from total protein extracts, indicating that only small chloroplast contaminants were present in our mitochondria preparations. Additionally, the signals from antibodies against AOX were strong in the mitochondria fractions (Fig. 6).

### Analysis of protein expression changes under salinity stress

**Fig. 7:**
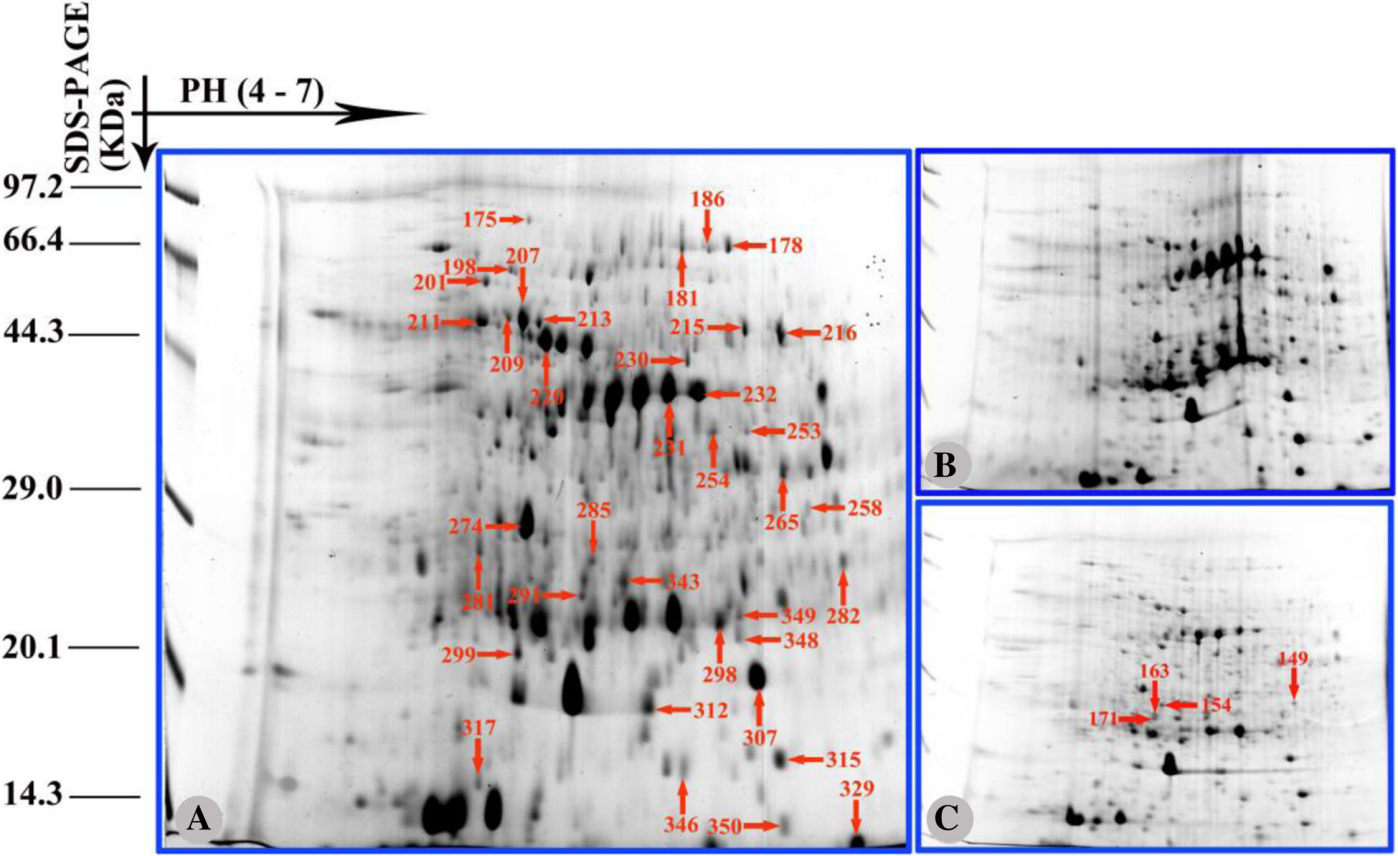
Coomassie Brilliant Blue (CBB)-stained two-dimensional electrophoresis gels of proteins in mitochondria of mesophyll cells of diploid (2×) *R. pseudoacacia* under 0, 250 mM and 500 mM NaCl treatment. Poteins were separated on 13 cm IPG strip (pH 4–7 linear gradient) usig isoelectric focusing, followed by sodium dodecyl sulfate polyacrylamide gel electrophoresis on a 12.5% gel. **a** 0 mM NaCl; **b** 250 mM NaCl; **c** 500 mM NaCl. The proteins information are listed in Table 2 and Additional file 1: Table S1

**Fig. 8:**
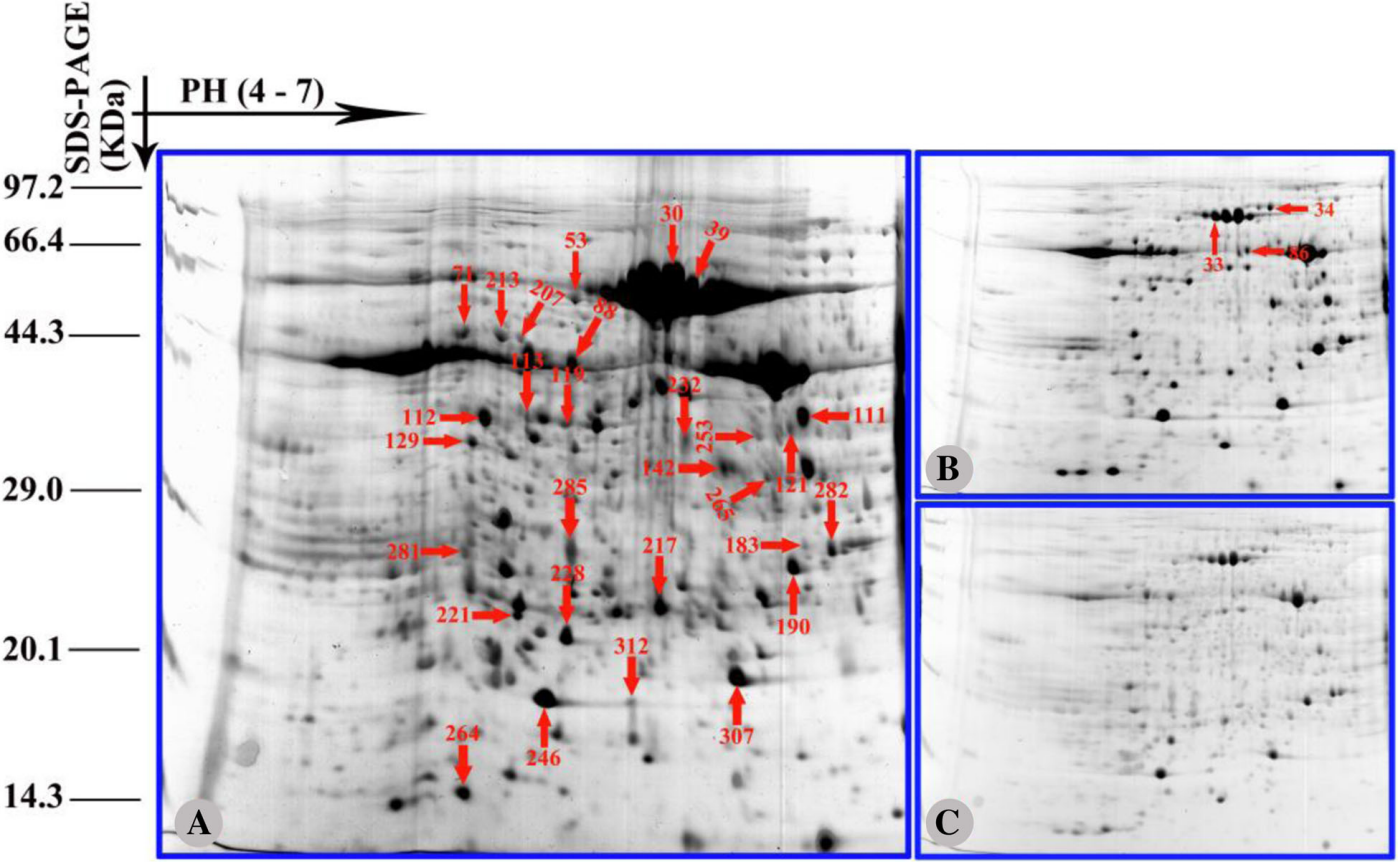
Coomassie Brilliant Blue (CBB)-stained two-dimensional electrophoresis gels of proteins in mitochondria of mesophyll cells of tetraploid (4×) *R. pseudoacacia* under 0, 250 mM and 500 mM NaCl treatment. Poteins were separated on 13 cm IPG strip (pH 4–7 linear gradient) usig isoelectric focusing, followed by sodium dodecyl sulfate polyacrylamide gel electrophoresis on a 12.5% gel. **a** 0 mM NaCl; **b** 250 mM NaCl; **c** 500 mM NaCl. The proteins information are listed in Table 2 and Additional file 1: Table S1

To investigate the changes in the expression profiles of mitochondrial proteins in 2× and 4× leaves under different salinity stress conditions, 2-DE analysis of leaf mitochondrial proteins from three biological replicates was carried out. Total mitochondrial protein from 2× and 4× leaves was extracted and purified. On the 2D gels for 2× and 4× mitochondria (Figs. 7 and 8), 144, 188, and 227 protein spots were detected from 2× samples under control conditions, 250 mM NaCl and 500 mM NaCl, respectively, and 207, 298, and 324 protein spots were detected from 4× samples under control conditions, 250 mM NaCl and 500 mM NaCl, respectively. Representative gels of 2× and 4× mitochondria are shown in Figs. 6 and 7, and the positions of differentially expressed spots are numbered. Only the protein spots with an absolute variation (≥ 2.5 fold with a *p*-value <0.05) according to quantitative image analysis were considered to be significantly changed after salt treatment and analysed further [52]. In total, 66 protein spots in both 2× and 4× mitochondria were compared using ImageMaster 2D Platinum 7.0 software (Amersham Biosciences, Piscataway, NJ, USA, 2011). Among the 66 differentially expressed protein spots, there were 32 spots from 2× mitochondria, 24 spots from 4× mitochondria and 10 spots from both 2× and 4× mitochondria showing significant changes in salt-treated plants compared to control plants. Overall, the number of changed proteins was higher in 2× than in 4× mitochondria, which may suggest that 2× mitochondria were more sensitive to NaCl stress. In 2× mitochondria treated with 500 mM NaCl, 15 proteins were up-regulated and 22 were down-regulated. In 2× mitochondria treated with 250 mM NaCl, 17 proteins were up-regulated and 17 were down-regulated. However, there were more up-regulated proteins (25 and 22) in 250 and 500 mM NaCl treated 4× mitochondria than in 2× mitochondria (Table 3).

### Functional classification of salinity stress-responsive proteins

**Fig. 9:**
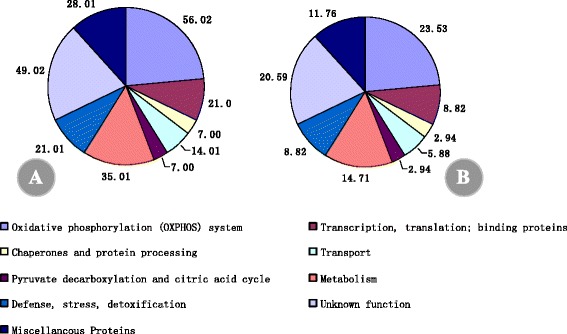
Functional classification of the identified proteins. The pie chart shows the distribution of the salt-responsive proteins into their functional classes in percentage. **a** Salt-responsive proteins in diploid (2×) *R. pseudoacacia*; **b** Salt-responsive proteins in tetraploid (4×) *R. pseudoacacia*

A total of 66 protein spots were excised from the gels and identified by mass spectrometry. The results of these analyses are listed in Additional file 1: Table S1. The tested proteins included 54 with a good match and 12 with an unknown function. Of the 54 matched proteins, 49 were of mitochondrial origin based on comparison to known mitochondrial proteins from other plants; however, 5 (spots 246, 258, 264, 307 and 312) were clearly identified as chloroplast contaminants. As the functional classification results show in Fig. 9, all proteins identified were assigned to 9 functional categories according to the proteome database: oxidative phosphorylation (OXPHOS) system (18); transcription, translation and DNA-binding proteins (12), chaperones and protein processing (4); transport (3); pyruvate decarboxylation and citric acid cycle (1); metabolism (6); defense, stress, detoxification (5), proteins of unknown function (12) and miscellaneous proteins (5). Here, these miscellaneous proteins were identified. The gene-ontology of some important proteins was carried out according to the results by DAVID web-server and KEGG method (Additional file 3: Table S3).

### Transcriptional expression of salinity stress-responsive genes

**Fig. 10:**
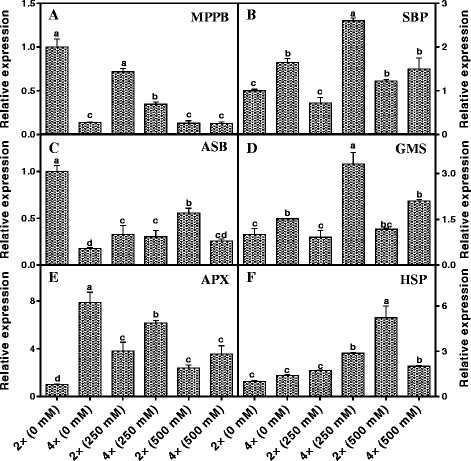
Expression of six salt stress related genes including cytochrome c reductase (complex III) mitochondrial processing peptidase subunit β, **a**; unknown protein gene that similar with sedoheptulose-1,7-bisphosphatase, SBP (**b**); ATP synthase (complex V) βsubnunit, ASB (**c**); glutamine synthetase, GMS (**d**); L-ascorbate peroxidase, APX (**e**) and heat shock protein HSP (**f**) of 2× and 4× black locust leaves after 7 days of treatment under 0, 250, and 500 mM NaCl, respectively. The genes are listed in Additional file 1: Table S1; Additional file 2: Table S2. Values represent the means of three separate experiments ± standard deviations (SD). Capital letters stand for significant difference at 5% level

**Fig. 11:**
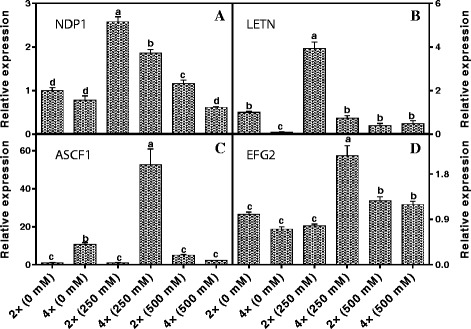
Expression of four salt stress related genes including NADH dehydrogenase (complex I) iron-sulfur protein 1, *NDP1* (**a**); lectin, *LETN* (**b**), ATP synthase (complex V)α subnunit *ASCF1* (**c**) and elongation factor G, *EFG2* (**d**) of 2× and 4× black locust leaves after 7 days of treatment under 0, 250, and 500 mM NaCl, respectively. The genes are listed in Additional file 1: Table S1; Additional file 2: Table S2. Values represent the means of three separate experiments ± standard deviations (SD). Capital letters stand for significant difference at 5% level

To investigate the relationship between the transcriptional and translational levels of genes responsive to salinity stress, we employed qRT-PCR to analyse 12 genes based on our proteomics data. Ten genes of the 12 selected were amplified successfully, but two genes, *NUO* (spot. 34) and *RPP8* (spot. 329) were not amplified (Additional file 4: Fig. S1). Among the ten successfully amplified genes, six were found in which the level of protein spots corresponded with their mRNA level, i.e., *MPPB* (spot. 215), *SBP* (spot. 129), *ASB* (spot. 220), *GMS* (spot. 113), *APX* (spot. 203), *HSP* (spot. 149) (Fig. 10). However, the other four genes showed different transcriptional expression trends compared with the protein expression pattern observed in the 2-DE assay, i.e., *NDP1* (spot. 178), *ASCF1* (spot. 213), *LETN* (spot. 217) and *EFG2* (spot. 175) (Fig. 11).

### Confirmation of the proteomic data

**Fig. 12:**
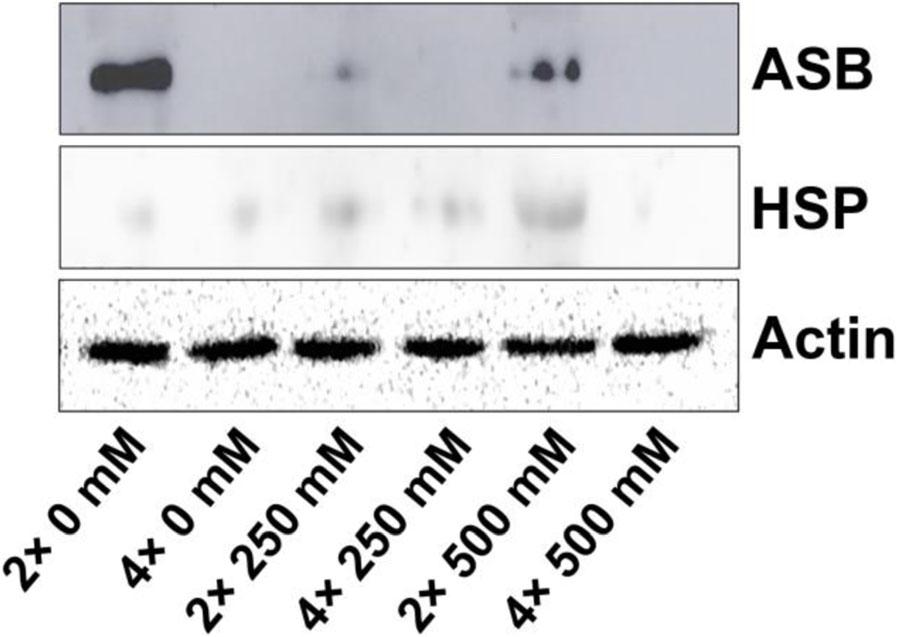
Western blot of ATP synthase β subnunit (ASB) and Heat Shock Protein 60 (HSP) and *β*-actin in mitochondrial protein fraction of 2× and 4× leaves

To confirm the proteomic data in our study, two plant specific antibodies against mitochondrial ATP synthase β subnunit (spot 220, ASB) and Heat Shock Protein 60 (spot 149, HSP) were used for immune-blot analysis against mitochondria protein. As the result shown in Fig. 12, there was a clear target band in 2× mitochondrial protein for ASB antibody, especially in control the signal is stronger than in 250 mM and 500 mM NaCl stressed 2×. Yet there was no signal for 4× mitochondrial protein samples. For Heat Shock Protein 60, there is a vigorous band in 500 mM NaCl stressed 2× mitochondrial protein, and the signal was relatively weak in other samples. The immune-blot result of ASB and HSP was similar with the proteome and qPCR data, which confirmed that ASB and HSP keep similar trend between their transcriptional and translational patterns in NaCl stress 2× and 4×, respectively.

## Discussion

### Physiological and biochemical characteristics and salt tolerance

The response of plants to salinity stress depends on the plant species and the severity of the salinity stress. In the present study, we investigated the responses of two black locust (*R. pseudoacacia*) species to salinity stress. The responses in leaf growth and physiological and biochemical characteristics to salinity stress were similar in the two species. In addition, some reports in *Populus* showed that vegetative growth traits, net photosynthetic rate (*P*n), relative chlorophyll content index (CCI), leaf area (LA), and whole-leaf photosynthetic efficiency (PEw) in polyploidy *Populus* were significantly higher than those in diploids, indicating that certain polyploid *Populus* groups had greater advantages in these respects [14]. Polyploid *Acacia senegal* also showed faster growth than their diploids [15], though, the reasons remain unclear. The relative effects of ploidy on mitochondrias between polyploid and diploid have not been systematically examined.

Salinity stress significantly inhibited leaf growth, damaged mitochondrial ultrastructure, increased MDA contents and respiration rates, and, at the same time, induced changes in antioxidant enzyme activities in the mitochondria of 2× and 4× leaves. In particular, H_2_O_2_ content was higher in 4× leaves in all conditions compared to that of 2× leaves (Fig. 3a) and was not changed with increase salinity stress, while, the H_2_O_2_ content in 2× leaves increased with salinity stress, especially in extreme conditions (500 mM NaCl). This may be because there is higher native H_2_O_2_ content in 4× leaves. Salinity stress did not appear to harm 4× leaves in our study. Accordingly, salinity stress induced no change in H_2_O_2_ content in 4× leaves, suggesting that 4× plants may be more tolerant to salinity stress than 2× plants. These results were also in accordance with the morphological data of 2× and 4× leaves under salinity stress (Fig. 1). To prevent cellular damage, H_2_O_2_ (a by-product of SOD activity) must be H_2_O through processing by APX, POD, and CAT, which regulate H_2_O_2_ levels in plants. In general, under salinity stress plants may generate abundant H_2_O_2_, a toxic species leading to lipid peroxidation and electrolyte leakage [53, 54]. However, some studies have shown that H_2_O_2_ may act as an effective signal molecule during stress responses to induce gene expression of antioxidant enzymes related to stress tolerance [55, 56]. This may be the reason we found increased H_2_O_2_ levels in the mitochondria of 2× leaves, especially under 500 mM NaCl (Fig. 3a). Increased accumulation of H_2_O_2_ has also been reported in isolated organelles after exposure to environmental stress [57, 58]. The increase in H_2_O_2_ in mitochondria after salt treatment indicated that their H_2_O_2_-detoxifying capacity did not match their generation of H_2_O_2_, as observed in chloroplasts of 2× leaves (data not shown). Similar results have also been reported in some previous studies [58]. In general, to control the level of H_2_O_2_ under stress conditions, plant mitochondria contain several enzymes that scavenge H_2_O_2_ and its precursor O_2_^−^, such as SOD, APX and GR [59]. The major H_2_O_2_ detoxification route is the ascorbate-glutathione cycle, which involves the antioxidant AsA, GSH, MDHAR, DHAR and GR in mitochondria [59]. In plants, AsA and GSH are crucial for biotic and abiotic stress tolerance [60]. Salinity stress can lead to osmotic stress, resulting in a higher accumulation of SOD, and the level of SOD accumulation is related to the level of salt tolerance [61], because SOD dismutates O_2_^−^ to H_2_O_2_ and O_2_. In the present study, NaCl treatment with 500 mM NaCl induced significant increases in SOD and APX activities in 4× mitochondria, while both enzyme activities were depressed in 2× mitochondria (Fig. 4c), demonstrating the marked improvement of salt tolerance in 4× plants. This increased ability to dismutate O_2_^−^ and detoxify H_2_O_2_ probably played a predominant role in the greater salinity tolerance of 4× mitochondria. Additionally, although 4× mitochondria had much lower MDHAR activity in the 0 mM and 250 mM NaCl treatments in comparison with 2× mitochondria, 4× mitochondria showed a similarly high MDHAR activity at 500 mM NaCl (Fig. 4f). This demonstrates that 4× mitochondria could acclimate to very high salinity. Concerning antioxidants, salt treatment caused a significant increase of AsA and GSH levels in 2× and 4× mitochondria (Fig. 5), but the absolute contents were greater in 4× mitochondria than in 2× mitochondria (Fig. 5) in each treatment, indicating that 4× mitochondria possessed more tolerance of, and greater ability to acclimate to, salinity stress.

Environmental stresses such as chilling, salinity stress and drought can cause visible injuries to plant mitochondria. In our previous study we observed that salinity stress resulted in plant mitochondrial damage [23], similar results were also observed in the present study (Fig. 2). Based on differences in mitochondrial ultrastructure between 2× and 4× samples under salinity stress, we found that 4× mitochondria showed more adaptability and tolerance to salinity stress compared with 2× mitochondria.

Salinity stress can bring about damage to mitochondrial membranes to plants, as assessed by lipid peroxidation (Fig. 3d). To escape or repair membrane damage caused by salinity stress, increased levels of antioxidants for maintaining membrane integrity can effectively inhibit lipid peroxidation, which can be measured by the level of MDA an indicator of membrane damage. In this study, MDA levels increased with the aggravation of salinity stress in both 2× and 4× mitochondria during the 7 days of stress, but the absolute content was greater in 2× than in 4× mitochondria (Fig. 3d), which suggests that 4× mitochondria could adjust to salinity stress more effectively than 2× mitochondria. APX, a component of the ascorbate-glutathione pathway, plays a key role in scavenging H_2_O_2_ [62, 63]. Lower levels of MDA (an end product of lipid perocidation) are associated with higher APX activity in salt tolerant tomato [64], rice [65] and sugar beet [66] plants, which was consistent with our results.

In this study, we also found that COX activity significantly changed in both 2× and 4× mitochondria after 250 mM NaCl treatment, which suggested that COX played an important role in the tolerance of plants to salinity stress. Generally, COX has been considered a mitochondrial marker enzyme, participating in an important pathway that is primarily used for ATP generation from ADP and inorganic P (Pi) through oxidative phosphorylation. Additionally, the activity of the COX pathway accounts for most of the respiratory O_2_ uptake and mitochondrial electron flux in plants, such that higher COX activity allows greater maximum electron flux [67]. Our results indicated that COX activity in 4× mitochondria was slightly higher than in 2× mitochondria after NaCl treatment (Fig. 4c), consistent with their difference in salt tolerance.

Leaf respiration rate increased under salinity stress in both species, reflecting the energetic demands of ion exclusion. Interestingly, 4× leaves showed a higher respiration rate compared to 2× leaves (Table 1). Although the relationship between growth and respiration rate is complex, perhaps high respiration rates under salinity stress could involve judicious allocation of stored carbon reserves to alleviate salinity stress [37]. For example, a higher respiration rate could contribute more to ion exclusion, since more energy can be produced by a higher respiration rate [68]. However, there are some exceptions [37]. Therefore, based on previous studies, there was no clear expectation of a respiratory rate response in relation to whole-plant salinity tolerance. However, our results obtained with 2× and 4× leaves add weight to the idea that increased respiration could enhance whole-plant salinity tolerance.

By analyzing changes in physiological and biochemical characteristics, we have uncovered some of the factors responsible for the greater salt tolerance of 4× leaves compared to in 2× leaves. Thus far, there are very strong links between mitochondrial antioxidant defences and whole-plant salinity tolerance, which were further measured using proteomic analysis to detect changes in defence-related mitochondrial proteins.

### Proteomic investigations

Intriguingly, in some cases the same proteins could each be found in two spots in 2× mitochondria: ATP synthase (complex V)α subunit (spots 209 and 213), ATP synthase (complex V)βsubunit (spots 211 and 220) (Table 2), and ATP synthase (complex V)α subunit (spots 53 and 213). ATP synthase (complex V)β subunit (spots 71 and 88) was also identified in two spots in 4× samples (Table 2). Further investigation revealed that these proteins had been identified from different plant species, and the p*I* and/or experimental *Mw* of the spots were different from their theoretical values. This might be explained by the presence of post-translational modifications such as phosphorylation, glycosylation and cleavage or different translational splicing, which may result in the alteration of protein charge and/or molecular mass [69, 70]. These same mitochondrial proteins showed different responses to salinity stress in 2× and 4× leaves. As an example, spots 53, 213, 209 and 213 were all identified as ATP synthase α subunit (ATPase), with an increasing trend under increased salinity of spots 53 and 213 in 4 × mitochondria, but a clear a decreasing trend of spots 209 and 213 in 2× mitochondria under increased salinity. Ten proteins including ATP synthase α subunit, chromosome segregation ATPases, myosin-like protein, ATP-dependent RNA helicase, kinesin heavy chain, heat shock protein, malate dehydrogenase and predicted protein were identified as altered under salinity stress in both 2× and 4× mitochondria. Surprisingly, the expression changes of these proteins differed between 2× and 4× mitochondria. For example, in 500 mM NaCl treated plant, heat shock protein (spot 282) was up-regulated in 4× mitochondria, but down-regulated in 2× mitochondria. These results suggest that different mechanisms might be triggered in 2× and 4× mitochondria in the presence of salinity stress.

**Table 2:** Differentially expressed proteins spots in mitochondria of mesophyll cells of diploid (2×) and tetraploid (4×) *R. pseudoacacia* using 2D–Gel Analysis under 0, 250 mM and 500 mM NaCl treatment

Spot no.^a^	Protein Name	Species	Accession^b^	Theor pI/KDa^c^	Exper pI/KDa^d^	Score^e^	SC (%)^f^	PN^g^	Relative V% ±Se^h^	
Oxidative phosphorylation (OXPHOS) system										
34	NADH-ubiquinone oxidoreductase (complex I) 75kDa subunit	*Zea mays*	gi|195648210	6.1/89.0	4.78/66	79	3%	2		T
178	NADH dehydrogenase (complex I) iron-sulfur protein 1	*Vitis vinifera*	gi|225453076	6.5/81.8	6.1/71	193	19%	14		D
190	putative NADH dehydrogenase (complex I)	*Bonyunia minor*	gi|6686959	6.0/5.18	6.53/27	580	18%	8		T
230	NAD^+^ binding site	*Glycine max*	gi|356525205	5.6/53.8	5.9/45	199	34%	26		D
291	family protein (complex I)	*Arabidopsis lyrata* L. subsp	gi|297312256	9.88/10.1	5.47/25	45	7%	9		D
215	Cytochrome c reductase (complex *III*) Mitochondrial processing peptidase subunit β	*Glycine max*	gi|356558971	6.27/58	6.2/50	399	29%	27		D
53	ATP synthase (complex V) α subnunit	*Arabidopsis thaliana*	gi|15219234	5.11/69	5.52/60	203	12%	5		T
71	ATP synthase (complex V) β subnunit	*Kalidium foliatum*	gi|118429132	4.93/54.1	5.02/52	264	13%	5		T
88	ATP synthase (complex V) β subnunit	*Stirlingia latifolia*	gi|3850914	5.09/52.8	5.51/45	548	31%	9		T
207	ATP synthase (complex V) α subnunit	*Manihot esculenta*	gi|169794058	5.13/55	5.1/52	733	35%	24		A
209	ATP synthase (complex V) α subnunit	*Vigna radiata*	gi|289066833	5.1/55	5.1/53	382	26%	24		D
211	ATP synthase (complex V) βsubnunit	*Glycine max*	gi|356575458	4.9/54	4.9/52	626	55%	33		D
213	ATP synthase (complex V) α subnunit	*Manihot esculenta*	gi|169794058	5.22/55	5.26/52	425	27%	20		A
220	ATP synthase (complex V) βsubnunit	*Glycine max*	gi|356536246	5.8/59	5.3/48	152	36%	21		D
223	ATP synthase (complex V) βsubnunit	*Glycine max*	gi|91214126	5.29/53	5.37/47	903	53%	31		D
198	Cell division protein ftsH	*Ricinus communis*	gi|255558698	6.43/75	5.1/64	258	35%	27		D
216	ATP_1_ gene product	*Lotus japonicus*	gi|372450305	6.01/55	6.44/50	509	45%	27		D
285	Chromosome segregation ATPases (Myosin-like protein)	*Medicago truncatula*	gi|357463145	4.65/206	5.5/27	52	14%	25		A
Transcription, translation; DNA-binding proteins										
33	pentatricopeptide repeat-containing protein	*Setaria italica*	gi|514771455	8.17/59.2	5.78/64	33	2%	1		T
171	Ankyrin repeat domain-containing protein	*Medicago truncatula*	gi|357500765	6.80/53	5.22/24	53	33%	12		D
175	Elongation factor G	*Vitis viniferad*	gi|359496425	5.52/85	5.2/86	328	41%	34		D
253	ATP-dependent RNA helicase	Brachypodium distachyon	gi|357126966	9.24/49	6.28/37	58	36%	12		A
181	zinc ion binding protein	*Arabidopsis thaliana*	gi|18399026	9.12/31	5.9/72	55	41%	10		D
298	Calcium-binding EF-hand family protein	*Theobroma cacao*	gi|508777222	4.82/16.5	6.14/23	31	4%	1		D
299	Calcium-binding protein	*Solanum lycopersicum*	gi|460378369	4.90/16.5	5.14/23	31	4%	1		D
232	kinesin heavy chain	*Ricinus communis*	gi|255537481	7.88/12	6.0/41	55	22%	18		A
315	50S ribosomal protein L21, mitochondrial	*Arabidopsis thaliana*	gi|29839556	5.52/30.9	6.4/17	43	30%			D
350	ribosomal protein S8	*Monomastix*	gi|224179526	10.1/16.4	6.46/14	29	8%	2		D
317	DNA binding protein, putative	*Ricinus communis*	gi|223531593	5.77/35.1	4.96/16	44	15%			D
343	pentatricopeptide repeat-containing protein	*Setaria italica*	gi|514771455	8.17/59.1	5.67/26	35	2%	1		D
Chaperones and protein processing										
282	Heat shock protein	*Medicago truncatula*	gi|357503161	5.10/71	6.75/27	118	22%	17		A
149	Heat shock protein	*Zea mays*	gi|162461165	7.88/26	6.3/26	57	35%	12		D
201	protein disulfide-isomerase	*Glycine max*	gi|356554621	5.28/56	5.0/62	47	9%	7		D
231	A1 cistron-splicing factor AAR2	*Arabidopsis thaliana*	gi|30697362	5.05/41	5.89/41	54	39%	12		D
Transport										
39	mitochondrial substrate carrier	*Volvox carteri f. nagariensis*	gi|300265179	6.25/36	6.06/61	43	11%	1		T
154	adenosine deaminase-like	*Glycine max*	gi|356521975	5.80/25	5.28/25	46	17%	8		D
339	eukaryotic translation initiation factor 2c	*Ricinus communis*	gi|223543695	9.29/110	6.04/14	47	3%	7		T
Pyruvate decarboxylation and citric acid cycle										
265	malate dehydrogenase	*Glycine max*	gi|373432589	5.91/35	6.45/33	147	40%	15		A
Metabolism										
86	Alanine aminotransferase	*Medicago truncatula*	gi|357485703	5.78/54.0	4.45/46	120	6%	2		T
113	Glutamine synthetase	*Ricinus communis*	gi|255551511	6.69/48.1	5.30/39	49	8%	1		T
119	Glutamine synthetase	*Canavalia lineata*	gi|6578120	6.28/47.6	5.46/39	112	9%	3		T
121	putative alcohol dehydrogenase	*Betula pendula*	gi|6723484	8.58/20.1	6.51/38	76	5%	1		T
142	P-loop containing nucleoside triphosphate hydrolases superfamily protein	*Arabidopsis thaliana*	gi|15219376	6.81/48.3	6.21/34	191	6%	2		T
346	fucosyltransferase CAZy family GT37-like protein	*Selaginella moellendorffii*	gi|300143173	7.91/58	5.97/16	40	20%	1		D
Defense, stress, detoxification										
111	1,3-β-glucan synthase subunit	*Fragaria vesca*	gi|470139357	8.99/204	6.57/39	41	4%	7		T
186	BZIP transcription factor	*Medicago truncatula*	gi|357486627	8.65/24	6.09/71	50	36%	11		D
329	disease resistance RPP8-like protein 3-like	*Glycine max*	gi|356538242	8.76/104	6.82/14	24	3%	1		D
203	L-ascorbate peroxidase	*Cicer arietinum*	gi|502145236	5.65/27.1	5.04/26	141	12%	2		T
217	lectin	*Astragalus falcatus*	gi|3819113	3.85/9.80	5.92/24	66	8%	1		T
Unknown function										
163	predicted protein	*Glycine max*	gi|168064083	9.36/27	5.23/24	49	48%	9		D
281	predicted protein	*Physcomitrella patens*	gi|168070396	6.56/47	4.9/28	54	31%	14		A
221	predicted protein	*Populus trichocarpa*	gi|224086409	10.8/14.4	5.26/24	42	8%	1		T
254	predicted protein	*Populus trichocarpa*	gi|224109480	5.31/47.2	6.11/37	65	17%	8		D
30	uncharacterized protein	*Zea mays*	gi|308082002	11.06/24.8	5.98/65	50	39%	8		T
349	uncharacterized protein	*Glycine max*	gi|359807168	6.24/32.7	6.24/24	384	22%	6		D
112	hypothetical protein	*Prunus persica*	gi|462400783	6.84/52	5.11/39	351	16%	6		T
183	hypothetical protein	*Musa acuminata* var. zebrina	gi|1491726	7.37/37.2	6.58/28	114	11%	3		T
348	hypothetical protein	*Vitis vinifera*	gi|147817756	5.54/33.8	6.23/22	148	24%	4		D
129	unknown	*Medicago truncatula*	gi|217073023	7.67/36.5	5.05/37	286	13%	4		T
228	unknown	*Lotus japonicus*	gi|388515001	5.74/21.5	5.48/22	148	12%	2		T
274	unknown	*Medicago truncatula*	gi|217071716	4.66/18.0	5.20/29	337	49%	5		D
Miscellancous Proteins										
246	Ribulose 1,5-bisphosphate carboxylase	*Loeseneriella*	gi|9909955	6.04/52.3	5.39/19	270	9%	4		T
258	Ribulose-1,5-bisphosphate carboxylase/oxygenase large subunit	*Ulmus alata*	gi|523330	6.05/53.1	5.86/16	315	12%	5		D
264	Ribulose bisphosphate carboxylase large chain	*Prostanthera nivea*	gi|671611	6.33/52.8	5.01/15	337	8%	5		T
307	Ribulose bisphosphate carboxylase large chain	Medicago truncatula	gi|357502811	6.13/53	6.33/20	235	21%	14		**A**
312	Ribulose-1,5-bisphosphate carboxylase/oxygenase large subunit	Lotus japonicus	gi|13518420	6.22/51	5.79/19	249	24%	15		**A**

^a^ Assigned spot number as indicated in Figs. 6 and 7. ^b^ Accession numbers according to the NCBIInr database. ^c^ Theoretical pI and masses (kDa) and of identified proteins. ^d^ Experimental pI and masses (kDa) and of identified proteins. ^e^ Mascot protein score reported after searching against the NCBInr database. ^f^ Sequence coverage. ^g^ Number of peptides sequenced. ^h^ Mean of relative protein abundance and standard error. D, Protein spots only detected in diploid black locust. T, Protein spots only detected in tetraploid black locust. A, Protein spots detected in diploid and tetraploid black locust. Six treatments including 1, 5, and 10 days after 500 mM NaCl treatment were performed

To further visualize these differentially regulated proteins, the identified proteins were classified into five groups according to their expression pattern (Tables 2 and 3). Patterns of differentially expressed proteins included (i) up-regulated proteins: proteins that were up-regulated under NaCl stress, (ii) down-regulated proteins: proteins that were down-regulated under NaCl stress, (iii) disappearing proteins: proteins that disappeared under NaCl stress, and (iv) visible proteins: proteins that were only detected in the NaCl treated samples. The expression levels of the identified proteins from 2× and 4× mitochondria displayed obvious differences. In 2× mitochondria treated with 250 mM NaCl, 17 spots were up-regulated and 15 spots were down-regulated, while in 4× mitochondria treated with 250 mM NaCl, 22 up-regulated spots and 7 down-regulated ones were observed compared to controls. Interestingly, although the number of differentially expressed protein spots in 4× mitochondria was smaller than in 2× mitochondria, under 500 mM NaCl stress more proteins were increased in 4× mitochondria (16 spots, compared to 14 in 2× mitochondria). Spots 149, 154, 163 and 171 were only visible in 2× mitochondria treated with 500 mM NaCl, which suggests that some proteins were induced by high salinity. Surprisingly, one spot (spot 346) disappeared after 250 mM NaCl treatment and four spots (spots 71, 129, 339 and 346) disappeared after 500 mM NaCl treatment, which may be linked to the difference in salinity stress tolerance of 2× and 4× plants.

**Table 3:** Number of protein spots significantly changed under different NaCl stress

	Upregulated spots	Downregulated spots	No significantly changed spots	Not detected spots	Newly detected spots
2×					
250 mM Vs 0 mM	17	17	5	3	0
500 mM Vs 0 mM	15	22	0	0	5
500 mM Vs 250 mM	14	20	0	0	8
Total	45	59	5	3	13
4×					
250 mM Vs 0 mM	25	8	0	0	1
500 mM Vs 0 mM	22	8	0	3	1
500 mM Vs 250 mM	11	20	0	3	0
Total	58	36	0	6	2

#### Oxidative phosphorylation (OXPHOS) related proteins

Oxidative phosphorylation is an important metabolic pathway in which mitochondria produce ATP using energy released by the oxidation of nutrients. In this study, 16 protein spots involved in OXPHOS were identified in both 2× and 4× mitochondria after salt treatment. In 4× mitochondria, four proteins (spots 34, 53, 88 and 190) were observed to increase in response to 250 mM and 500 mM NaCl treatment. Up-regulation of oxidative phosphorylation related proteins can ensure adequate ATP for other metabolic processes under salinity stress [71], which is partly supported by our findings. For example, an increased abundance of metabolism-related proteins involved in glutamine metabolism (spots 113 and 119) and alanine metabolism (spot 86) in 4× mitochondria is listed in Table 2. Glutamine synthase, GS (EC 6.3.1.2), is a particularly important enzyme for nitrogen metabolism. Nitrogen, a key element for plant growth and reproduction, is an essential building block of nucleic acids and proteins [72]. Leaf GS activity is known to be increased by salinity stress in cashew leaves [73]. Similarly, GS showed different levels in leaves of wheat (*Triticum aestivum*) seedlings exposed to different salinity [74]. In the present study, the increase of GS may contribute to the accumulation of nitrogen, promoting the synthesis of nucleic acids and proteins and, thereby, increased plant growth. In accordance with our results, JAG Silveira, RDA Viégas, IMAD Rocha, RDA Moreira and JTA Oliveira [73] and CO Silva-Ortega, AE Ochoa-Alfaro, JA Reyes-Agüero, GA Aguado-Santacruz and JF Jiménez-Bremont [75] observed that salinity stress increased GS activity. Clearly, these results indicate that GS plays important roles in response to salinity stress.

In contrast to the case of 4× mitochondria, oxidative-phosphorylation-related proteins such as NADH dehydrogenase (complex I, spot 178), NAD^+^ binding site (spot 230), COX (complex III, spot 215) and ATP synthase (complex V, spots 209, 211 and 220) in 2× mitochondria showed a significant reduction under NaCl stress. In plants, these four proteins are essential for mitochondrial electron transport. The mitochondrial electron transport chain (ETC) is a major site of ROS production; excessive ROS can lead to oxidative stress [76]. When proteins involved in mitochondrial electron transport are down-regulated during biotic or abiotic stress, ROS production may be enhanced to a level where mitochondria may be damaged by oxidative stress [76]. In this study, we found that SOD, APX, MDHAR and GR all increased to cope with oxidative stress in 4× plants under salinity stress. By contrast, increased ROS production associated with down-regulation of OXPHOS-related proteins in 2× plants was not counteracted by a sufficient increase in anti-oxidative enzyme activities.

#### Transcription, translation and DNA-binding proteins

According to previous studies, DNA-transcription, translation and binding proteins with low abundance in plant mitochondria have been poorly represented [77]. In our study, there were 12 proteins in the transcription, translation and DNA-binding proteins category (Table 2). Two pentatricopeptide repeat (PPR) proteins were identified: spots 33 and 343. Even with only two identified PPR proteins, there were clear differences between 2× and 4× mitochondria. PPR proteins decreased in abundance with increasing concentrations of NaCl in 4× mitochondria, while the opposite change occurred in 2× mitochondria. PPRs are involved in RNA metabolism and post-transcriptional regulation in plant organelles [78]. Though extensive studies have been performed on PPRs, their functions remain largely unknown [79]. In 2× mitochondria after salt treatment, elongation factor, ribosomal protein and a series of binding proteins were also identified. To our knowledge, these proteins are involved in plant salinity stress signalling [80–82]. Most of these proteins (spots 298, 299, 315, 350 and 317) reached maximal levels after 250 mM NaCl treatment, and were down-regulated to lower levels after 500 mM NaCl treatment. These results indicate that 2× plants were capable of coping with relatively low salt conditions by enhancing protein expression, but were unable to just to higher salinity due to signalling inhibition.

#### Chaperones and protein processing

In this study, one contrasting result observed was the clear induction of heat shock protein 70 (HSP70) in 4× mitochondria after NaCl treatment, whereas no clear induction was observed in salt-stressed 2× mitochondria (spot 282). This induction is consistent with many reports highlighting relationships between HSP70 and plant programmed cell death (PCD) [83, 84] and HSP70 induction under environmental stresses in plants [33, 85]. Interestingly, we also observed an increase in one small heat shock protein (spot 149) in salt-treated 2× mitochondria. This was not unexpected considering that 2× black locust is known to possess some level of salt tolerance. A protein disulfide-isomerase (spot 201), which acts as a protein-folding catalyse that interacts with nascent polypeptides to catalyse the formation, isomerisation, and reduction or oxidation of disulfide bonds was down-regulated after NaCl treatment of 2× leaves. Protein disulfide-isomerase (PDI) is known to be induced under cold, salt and abscisic acid (ABA) stresses [86]. In addition, in 2× plants, the A1 cistron-splicing factor AAR2, which is involved in splicing pre-mRNA of the a1 cistron and other genes that are important for cell growth appeared to decrease in abundance after 500 mM NaCl treatment. Taken together, these results indicate possible inhibitory effects of salinity stress on protein processing in 2× mitochondria.

#### Defense-responsive proteins

Salinity stress can induce the production of ROS, which may cause damage to plant cells and also act as secondary messengers [87]. To regulate ROS level, many defence proteins are induced during plant responses to biotic or abiotic stresses [88]. However, their response mechanisms are not well known. In the present study, five defence-related proteins including BZIP transcription factor (spot 186), disease resistance RPP8-like protein 3-like (spot 329), L-ascorbate peroxidase (spot 203) and lectin (spot 217) were found to be differentially regulated after salt treatment. In 4× mitochondria, the L-ascorbate peroxidase protein was down-regulated, which requires further investigation since APX activity itself was increased. In 4× mitochondria, lectin levels were increased by salinity stress. The differential expression of defence-related proteins may help to enhance the tolerance of 4× mitochondria to salinity stress and decrease the risk of damage. Disease resistance RPP8-like protein 3-like (spot 329) belongs to the disease resistance NB-LRR family, and it may play a crucial role in maintaining redox homeostasis under various stresses [89, 90]. Our results show that disease resistance RPP8-like protein 3-like was expressed at lower levels in salt-stressed 2× mitochondria. The down-regulation of this RPP8-like protein may be associated with susceptibility of 2× plants to salinity stress. Our results also imply different response mechanisms in the defence system in different ploidy groups during acclimation to salinity stress.

#### Membrane transport and citric acid cycle

The main family of proteins in mitochondrial stress response is the mitochondrial substrate carrier protein family (MCF) [91]. In our study, only one MCF protein (spot 39) was found and it was down-regulated in 4× mitochondria after salinity stress. However, no MCF proteins were detected in 2× mitochondria. These results suggest that tolerant plants can respond quickly under salinity stress.

One protein (spot 265) related to the citric acid cycle was detected in both 2× and 4× mitochondria after NaCl treatment. However, spot 265 increased with salinity in 4× mitochondria, while it was significantly down-regulated in 2× mitochondria under increasing concentrations of NaCl.

#### Proteins of unknown function

A total of 13 proteins were identified as proteins of unknown function. Among these proteins, only one (spot 281) was induced in both 2× and 4× mitochondria after NaCl treatment. The accumulation of this protein suggested that this protein may be of particular importance in protecting plants from damage caused by salinity stress. In 4× mitochondria, three unknown proteins (spots 30, 112 and 221) increased in expression after salt treatment, but spots 129, 183 and 228 decreased in expression after salt treatment. We proposed that these unknown proteins might act as signalling molecules, although there is a great difference in their abundance. In 2× mitochondria, two proteins (spots 163 and 154) were induced only under 500 mM NaCl treatment. These findings suggest that we should pay attention to function of these proteins in plant tolerance to salt in a future study. In addition, no Arabidopsis orthologues of these unknown proteins have been identified in databases (Table 2). Nevertheless, our results indicate conservation of a number of unknown functional proteins from other plant species, thereby providing a clue for identification of novel mitochondrial functions in plants [92].

In addition to the functional mitochondrial proteins identified above, 5 miscellaneous proteins were detected by MS analysis (spots 246, 258, 264, 307 and 312 in Table 2). Slight contamination of the mitochondrial fraction by chloroplast proteins was visible under all purification conditions, because of the high degree of overlap between mitochondria and chloroplasts based on their density and size [93, 94]. Thus, the isolated mitochondria may be non-specifically mixed with chloroplast proteins. The methods for extraction and purification of mitochondrial protein from plant leaves remain to be improved.

### Transcriptional investigations

To correlate the levels of the identified salinity stress response-related proteins with their gene expression changes, qRT-PCR was employed to analyse the transcription levels of 12 genes (Additional file 2: Table S2). Six of the expressed mRNA levels them showed similar trends to their protein expression pattern under salinity stress (Fig. 10). This consistency suggested that these proteins may be initially regulated at the transcriptional level after salt treatment and/or they might induce related signal transduction pathways to resist salinity stress. In contrast, four genes showed different or reversed trends when comparing mRNA levels and protein expression (Fig. 11). This inconsistency between the protein and transcription levels could be due to post-translational processing or post-transcriptional regulation (47, 48). On the other hand, the parallel and independent changes between protein and mRNA levels for these four genes might reflect the complex regulatory mechanisms of plants responding to salinity stress. This inconsistency has also been found by some previous studies (49, 50, 51). Unfortunately, two genes, *NUO* (spot. 34) and *RPP8* (spot. 329) had no PCR results.

In addition, four genes showed different or reverse trends with their protein expression (Fig. 11). These four genes have effective positive role in regulating the tolerance of salinity stress. For example, NADH dehydrogenase, complex I (spot. 178) located in the inner mitochondrial membrane can contain type II NDH that by pass complex I and supply electrons to the ubiquinone pool [95]. Generally, a stimulation of O_2_^•−^ generation dependent on NADH-and succinate has been reported in plants under salinity, with a higher increase insensitive cultivars than in tolerant plants [96]. In particular, three subunits of the ATP synthase CF1, α (spot. 213), β and γ were affected by salt treatment in many plants [97]. Currently, there is growing evidence that the ATP synthase is also a target for a damaging effect caused by salt stress [98]. This change can protect the plant from further damage [97]. Another protein, lectins (spot. 217) residing in the nucleocytoplasmic compartment are known to be implicated in biotic and abiotic stress responses [99]. As part of the plant’s immune system, lectins can also act as immune receptors and/or defense proteins [100]. Additionally, as a molecular chaperone, elongation factor G (EF-Tu protein, spot 175) can protect other proteins from thermal aggregation and degradation, which plays a role in the elongation phase of protein biosynthesis and has been studied in several plants in response to abiotic stress, such as extreme salinity, temperatures and drought [101, 102].

## Conclusions

To date, there have been few reports concerning the relationship between mitochondrial function and salt tolerance. To gain a comprehensive physiological and biochemical and proteomic understanding of mitochondrial salinity tolerance in 2× and 4× *R. pseudoacacia*, we compared physiological and comparative proteomic traits of mitochondria in leaves of 2× and 4× after salinity stress. Unlike 4× mitochondria, 2× mitochondria showed significant stress symptoms in saline condition, resulting in wilting leaves, high MDA and H_2_O_2_ levels, decreased leaf respiration rate and inefficient defence systems involving antioxidant enzymes and antioxidants. In addition, defence-related proteins, metabolism-related proteins, heat shock proteins, membrane transport proteins and citric acid cycle-related proteins in 4× leaves were also induced by high salinity. These up-regulated proteins implied that defence, metabolism, ROS scavenging and protein transport may work cooperatively to maintain mitochondrial homeostasis in 4× under salinity stress. Accordingly, it can be reasonably expected that salt tolerance mechanisms in 4× are sophisticated. In contrast, the identification of salt-responsive proteins in 4× that were different from those in 2× (e.g., processing, binding and oxidative-phosphorylation-related proteins) (Additional file 1: Table S1; Additional file 2: Table S2) may provide new insights into the molecular mechanisms of resistance to salinity stress.

**Fig. 13:**
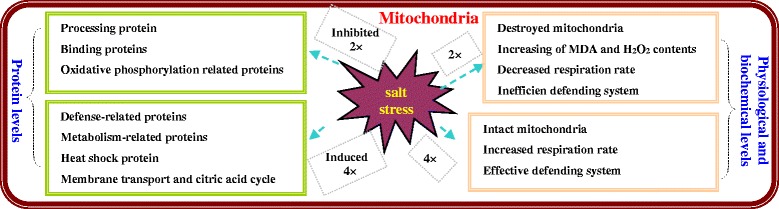
Potential model of the salt stress response of the physiological and biochemical and related-proteins between diploid (2×) and tetraploid (4×) *R. pseudoacacia*

Thus, we proposed a potential mechanism for the salinity tolerance of tetraploid black locust based on physiological and biochemical characteristics and related-proteins, which is presented in Fig. 13. In this pathway, a salinity stress signal (H_2_O_2_) induces signal perception, followed by the generation of antioxidants including AsA and GSH and antioxidant enzymes including APX, GR and MDHAR. These signals further interact with major stress responsive proteins. In our study, the abundance of many protein species involved in this proposed pathway was altered under salinity stress (Fig. 12), suggesting that these proteins function in response to salinity stress in both 2× and 4×. Here, mitochondria of 4× can perceive salinity stress signals through increasing the respiration rate, regulating the cellular defence system, maintaining the stability of mitochondrial membranes and regulating the levels of proteins related to defence, metabolism, membrane transport and the citric acid cycle to achieve mitochondrial homeostasis after salt treatment. Although polyploidy events in tetraploid black locust are believed to be responsible for increasing the size of plant organs and enhancing tolerance to environmental stresses, thorough transcriptomics studies have not been performed, and the relevant genes have not all been revealed, so the molecular and biological mechanisms regulating the major characteristics and the effects of polyploidy in tetraploid black locust remain unknown. Thus, in the future, transcriptomics and metabolomics should be used to gain a systematic and comprehensive understanding of the molecular networks of polyploid plants under salinity stress.

## Additional files

Additional file 1: Table S1. Differentially expressed proteins spots in mitochondria of mesophyll cells of diploid (2×) and tetraploid (4×) *R. pseudoacacia* using 2D–Gel Analysis under 0, 250 mM and 500 mM NaCl treatment. (XLS 16278 kb)

Additional file 2: Table S2. The primer sequences for real time PCR. (DOCX 17 kb)

Additional file 3: Table S3. The information of different expressed proteins according to ID using DAVID web-server. (XLS 46 kb)

Additional file 4: Figure S1. Semi-quantitative PCR analysis of ten genes including *HSP* (heat shock protein), *MPPB* (cytochrome c reductase (complex III) mitochondrial processing peptidase subunit β), *LETN* (lectin), *EFG2* (elongation factor G), *NDP1* (NADH dehydrogenase (complex I) iron-sulfur protein 1), *APX* (L-ascorbate peroxidase), *GMS* (glutamine synthetase), *ASB* (ATP synthase (complex V) β subunit), *ASCF* (ATP synthase (complex V) α subunit), *SBP* (an unknown protein gene that similar with sedoheptulose-1,7-bisphosphatase) and β-actin of 2× and 4× black locust leaves after 7 days of treatment under 0, 250, and 500 mM NaCl, respectively. DCK, 2× under 0 mM NaCl; D250, 2× under 250 mM NaCl; D500, 2× under 500 mM NaCl; TCK, 4× under 0 mM NaCl; T250, 4× under 250 mM NaCl; T500, 4× under 500 mM NaCl. (DOCX 246 kb)
