# Supplementary material for: Comparative mitochondrial proteomic, physiological, biochemical and ultrastructural profiling reveal factors underpinning salt tolerance in tetraploid black locust (Robinia pseudoacacia L.)
Source: BMC Genomics. 2017 Aug 22;18:648. doi: 10.1186/s12864-017-4038-2 (PMC5568289; doi:10.1186/s12864-017-4038-2)
Supplement: Supplementary file 2 — The primer sequences for real time PCR. (DOCX 17 kb) [file 12864_2017_4038_MOESM2_ESM.docx]

Supplemental Table 2: The primer sequences for real time PCR

| Spot No. | Gene name | Forward (5’-3’) | Reverse (5’-3’) | Tm | Cycle  number | Product length (bp) |
| --- | --- | --- | --- | --- | --- | --- |
|  | *ACTIN* | TCCATAATGAAATGTGATGTTG  GCCCAATTTGTGATCAGG | GGAAGGTGCTTAGAGATGC  TGTACATTGAATGCACCG | 53 | 35 | 217 |
| 178 | NADH dehydrogenase (complex I) iron-sulfur protein 1 (*NDP1*) |  |  | 52 | 40 | 158 |
| 215 | cytochrome c reductase (complex Ⅲ) mitochondrial processing peptidase subunit β(*MPPB*) | TCCCACCACAGCTTCTCA | CAAATTGTGCAAGGGGAA | 52 | 40 | 98 |
| 213 | ATP synthase (complex V)α subnunit *(ASCF1*) | TGTCTCTTCTATTACGAAGACC | CAGCTACTTGTTTCATGGCT | 54 | 35 | 321 |
| 217 | Lectin *(LETN)* | GTTGCAGTTGAATTTGACAC | GAAACCAACTCTTACCCACT | 51 | 40 | 267 |
| 220 | ATP synthase (complex V) β subnunit (*ASB*) | GACAGATCCTGCTCCTGC | CATCCAACACACCCTGGA | 57 | 35 | 389 |
| 175 | elongation factor G (*EFG2*) | TGGACTTCCCTGATCCTG | TGGAAAGAAGGGTCTTCC | 55 | 40 | 112 |
| 113 | glutamine synthetase (*GMS*) | TATCCTGGTCCTCAGGGTC | GCCCAGATGTGATCACCA | 57 | 40 | 142 |
| 129 | sedoheptulose-1,7-bisphosphatase (*SBP*) | TTGATGAAGGAAAATGGCA | GGCACCATTCCTCCAGTG | 51 | 40 | 169 |
| 203 | L-ascorbate peroxidase (*APX*) | GTTTGGCATGGCACTCTG | GTGGTGGCTCAGGCTTGT | 56 | 35 | 267 |
| 149 | Heat shock protein (*HSP*) | AGGAGGTGTCTCATGAGTGG | GGCTTCTTCCTTGTGTCAAA | 55 | 35 | 232 |
| 34 | NADH-ubiquinone oxidoreductase (*NUO*) | TGATGGGTGCTGATGATG | CCAACTGTTGGAACTGCA | 52 | 45 | 196 |
| 329 | disease resistance RPP8-like protein 3-like (*RPP8*) | GGGAAAACCACTTTGGCT | CCCCAAATGTCATCAAGC | 53 | 45 | 248 |
