# Supplementary material for: Comparative mitochondrial proteomic, physiological, biochemical and ultrastructural profiling reveal factors underpinning salt tolerance in tetraploid black locust (Robinia pseudoacacia L.)
Source: BMC Genomics. 2017 Aug 22;18:648. doi: 10.1186/s12864-017-4038-2 (PMC5568289; doi:10.1186/s12864-017-4038-2)
Supplement: Supplementary file 4 — Semi-quantitative PCR analysis of ten genes including HSP (heat shock protein), MPPB (cytochrome c reductase (complex III) mitochondrial processing peptidase subunit β), LETN (lectin), EFG2 (elongation factor G), NDP1 (NADH dehydrogenase (complex I) iron-sulfur protein 1), APX (L-ascorbate peroxidase), GMS (glutamine synthetase), ASB (ATP synthase (complex V) β subunit), ASCF (ATP synthase (complex V) α subunit), SBP (an unknown protein gene that similar with sedoheptulose-1,7-bisphosphatase) and β-actin of 2× and 4× black locust leaves after 7 days of treatment under 0, 250, and 500 mM NaCl, respectively. DCK, 2× under 0 mM NaCl; D250, 2× under 250 mM NaCl; D500, 2× under 500 mM NaCl; TCK, 4× under 0 mM NaCl; T250, 4× under 250 mM NaCl; T500, 4× under 500 mM NaCl. (DOCX 246 kb) [file 12864_2017_4038_MOESM4_ESM.docx]

Figure. S1. Semi-quantitative PCR analysis of ten genes including *HSP* (heat shock protein), *MPPB* (cytochrome c reductase (complex Ⅲ) mitochondrial processing peptidase subunit β), *LETN* (lectin), *EFG2* (elongation factor G), *NDP1* (NADH dehydrogenase (complex I) iron-sulfur protein 1), *APX* (L-ascorbate peroxidase), *GMS* (glutamine synthetase), *ASB* (ATP synthase (complex V)βsubunit), *ASCF* (ATP synthase (complex V)α subunit), *SBP* (an unknown protein gene that similar with sedoheptulose-1,7-bisphosphatase) and β-actin of 2× and 4× black locust leaves after 7 days of treatment under 0, 250, and 500 mM NaCl, respectively. DCK, 2× under 0 mM NaCl; D250, 2× under 250 mM NaCl; D500, 2× under 500 mM NaCl; TCK, 4× under 0 mM NaCl; T250, 4× under 250 mM NaCl; T500, 4× under 500 mM NaCl.
